# Optimization and Validation of Universal Real-Time RT-PCR Assay to Detect Virulent Newcastle Disease Viruses

**DOI:** 10.3390/v17050670

**Published:** 2025-05-03

**Authors:** Ellen Ruth Alexander Morris, Megan E. Schroeder, Phelue N. Anderson, Lisa J. Schroeder, Nicholas Monday, Gabriel Senties-Cue, Martin Ficken, Pamela J. Ferro, David L. Suarez, Kiril M. Dimitrov

**Affiliations:** 1 Texas A&M Veterinary Medical Diagnostic Laboratory, College Station, TX 77843, USA; ellenruth.alexander@tvmdl.tamu.edu (E.R.A.M.); megan.schroeder@tvmdl.tamu.edu (M.E.S.); pj.ferro87@gmail.com (P.J.F.); 2 Texas A&M Veterinary Medical Diagnostic Laboratory, Center, TX 75935, USA; phelue.anderson@tvmdl.tamu.edu (P.N.A.); carlos.senties-cue@tvmdl.tamu.edu (G.S.-C.); 3 Texas A&M Veterinary Medical Diagnostic Laboratory, Gonzales, TX 78629, USA; lisa.schroeder@tvmdl.tamu.edu (L.J.S.); marty.ficken@tvmdl.tamu.edu (M.F.); 4 Texas A&M Veterinary Medical Diagnostic Laboratory, Canyon, TX 79016, USA; nick.monday@ag.tamu.edu; 5 Exotic and Emerging Avian Viral Disease Research Unit, Southeast Poultry Research Laboratory, U.S. National Poultry Research Center, U.S. Department of Agriculture, Athens, GA 30605, USA; david.suarez@usda.gov

**Keywords:** APMV-1, Newcastle disease, rRT-PCR, qPCR, genotype, NDV, avian paramyxovirus 1, exogenous internal positive control

## Abstract

Newcastle disease, caused by virulent strains of avian paramyxovirus 1 (APMV-1), occurs globally and has significant social and economic impact. APMV-1 is a rapidly evolving RNA virus and is genetically divided into class I and class II with almost all virulent viruses being of class II. The considerable genetic diversity of the virus adds complexity to maintaining the high sensitivity and specificity of molecular detection assays. The current USDA’s fusion gene rRT-PCR assay was designed for class II APMV-1 isolates with an emphasis on early-2000s US strains. Assessment with globally circulating genotypes confirmed previously described lower sensitivity (sub-genotypes VII.1.1, VII.2) and identified absence of detection (genotype XIV). An additional forward primer and two probes were designed using a comprehensive complete fusion gene sequence database. The optimized multiplex assay detected genotype XIV and improved sensitivity for sub-genotypes VII.1.1 and VII.2, with maintained sensitivity for the remaining genotypes. No near-neighbors or APMV-1 of low virulence were detected. Using field and experimental clinical samples, both the specificity and sensitivity were determined to be 100%, compared to the current assay with 100% and 93%, respectively. The new assay identifies all known chicken virulent APMV-1 genotypes with the benefit of using an exogenous internal positive control, which monitors extraction efficiency and inhibitors.

## 1. Introduction

Newcastle disease (ND) is caused by virulent strains of avian paramyxovirus 1 (APMV-1) and significantly impacts poultry industries worldwide [1,2]. APMV-1 has been renamed multiple times during the last decade with the current name being *Orthoavulavirus javaense* [3]; however, for the purpose of this work, the widely known APMV-1 will be used. APMV-1 has high genetic diversity and two classes, class I and class II, which encompass at least 22 genotypes. The majority of the virulent viruses belong to class II. Severe and often fatal infections with virulent APMV-1 are seen in chickens, especially in naïve flocks, and the identification of virulent APMV-1 may result in trade restrictions for exporting countries [4]. However, low-virulence APMV-1 strains are endemic in wild birds and are commonly found in poultry, including widely used attenuated vaccine strains. Current vaccines are widely implemented among poultry operations and help mitigate disease but do not prevent infection with subsequent viral shedding [5,6]. Even with the implementation of efficient vaccines, annual outbreaks occur in Asia, South America, and Africa [1,7,8], while occasional outbreaks are documented in Europe and North America [9,10]. With its worldwide presence, APMV-1 causes significant economic losses and can impact food and economic security, particularly for small-holder farmers. In countries with a developed poultry industry, outbreaks can be costly to control, including the recent example of the ND outbreak in California during 2018–2019 impacting at least 476 poultry premises. The rapid, sensitive, scalable, and accurate detection of virulent APMV-1 strains using methods such as real-time reverse transcriptase PCR (rRT-PCR) coupled with a rapid response, including the depopulation of infected flocks, can be the most efficient approach to eradicate the virus in countries normally free of infection.

The detection of both low-virulence and highly virulent APMV-1 can be achieved by using primers designed to conserved targets including the nucleoprotein (NP), matrix (M), fusion (F), and large RNA polymerase (L) proteins. The most widely utilized assay to detect all APMV-1 targets the M-gene [11], but alternative assays targeting the NP and L genes have also been reported [12]. Molecular diagnostics play an important role in confirming a clinical diagnosis, and multiple conventional RT-PCR [12,13,14,15] and rRT-PCR [11,16,17,18,19] protocols are published. The use of rRT-PCR assays has become the de facto standard because they are more scalable to respond to an outbreak with increased testing requirements and have lower labor requirements with less opportunity for cross contamination of samples. In the US, ND is a regulated disease, and for it to be an official test in the National Animal Health Laboratory Network (NAHLN) of laboratories, the assay must be approved by the USDA. The current USDA testing workflow includes screening for all APMV-1 strains with an rRT-PCR assay targeting the M-gene, and then predicting the virulence of APMV-1 using a fusion (F)-gene assay [11]. The fusion cleavage site is the main molecular determinant of APMV-1 virulence, and a clear pattern of amino acids can be used to predict whether a virus meets the standard of a virulent and reportable APMV-1 [11]. The F-gene assay targets the fusion cleavage site, and the rRT-PCR probe differentiates between virulent and low-virulence APMV-1 isolates based on how well the probe anneals to the target. Testing APMV-1 isolates from different sub-genotypes circulating with the currently validated USDA F-gene assay has resulted in several published [20,21,22] or unpublished reports (data generated by the USDA’s Southeast Poultry Research Laboratory [SEPRL] in studies of virulent APMV-1 from Kenya, Colombia, and Pakistan) of either lower sensitivity or false negatives. Other rRT-PCR assays have tried to improve upon the current assay [11,23] by adding several indeterminate bases to the primers and probe set [16]. This approach did not account for all the currently circulating sub-genotypes, but does integrate the pigeon sub-genotypes, VI and XXI. While some investigators describe using SYBR green with a primer set designed to detect mesogenic (of moderate virulence for chickens) and velogenic (of high virulence for chickens) APMV-1 strains [24], other assays also use the MGB labeled probe but do not address the detection of all the contemporary APMV-1 [2,17,18].

Since the current USDA-approved assays were designed (in response to the 2002–2003 California outbreak), new APMV-1 variants have emerged in Asia, the Middle East, Africa, Europe, and Central and South America [10,25,26]. With global air travel and the legal and illegal movement of birds throughout the world, any of these actively circulating viruses pose a constant threat to the poultry industries. The continuous evolution of these viruses presents diagnostic challenges as target-oriented assays might fail to detect new and emerging variants [27,28]. Due to the increased sequencing efforts worldwide and the advances of next-generation sequencing, more genomic data are available on circulating APMV-1 genotypes. This provides an opportunity to perform thorough analyses of APMV-1 genetic diversity and optimize existing assays to detect all circulating genotypes. This improved capability for rapid detection will enhance the ability to accurately identify virulent APMV-1.

The goal of this study was to evaluate and improve the current USDA-approved rRT-PCR assay for the detection of virulent APMV-1. A dataset of all publicly available fusion gene sequences of all APMV-1 genotypes was utilized to perform comprehensive in silico and SNP analyses, and design new primers and probes. The newly designed oligonucleotides—as well as an exogenous internal positive control (XIPC) that aids the verification of effective nucleic acid purification and identification of false negatives due to PCR inhibitors [29]—were added to the existing protocol, resulting in a multiplex assay. Testing of the new multiplex assay was performed using representative viruses from each genotype. Finally, this new assay was evaluated across several different laboratories using a wide variety of sample types, including those obtained from clinical and experimental cases and from field submissions. The generated improved F-gene rRT-PCR multiplex assay will enhance laboratories in their capacity of rapid and early detection of virulent APMV-1 circulating worldwide and aid in the prevention of or response to outbreaks.

## 2. Materials and Methods

### 2.1. Fusion Sequence Database Generation and Curation

Complete F-gene coding sequences were downloaded from NCBI GenBank [30]. Metadata such as isolate name, host, year, and location of isolation were all recorded when available [2]. All sequences were aligned using Multiple Alignment with Fast Fourier Transformation (MAFFT) [31] as implemented in Geneious Prime 2022.2.2 (https://www.geneious.com). Sequences were translated to amino acids to establish virulence status using the cleavage site motif. Low virulence was established by a lack of multiple basic amino acids between position 113–116 and leucine at position 117. Conversely, virulent viruses had multiple basic amino acids between position 113–116 and a phenylalanine at position 117 [32]. Sequences identified as 100% identical were removed, and only a single representative was retained. Each sequence in the alignment was evaluated and all gaps and insertions that cause alignment shifts were deleted. Sequences that were found to originate from man-made clones, mutant viruses, and chimeric viruses were removed from the dataset. Sequences that were identified as spillovers or escapes (recent viruses identical or almost identical to standard strains originally isolated decades before) were also excluded from further analyses. In addition, all remaining sequences were subjected to recombination analysis using the RDP4 program, and sequences with recombination events were removed. A phylogenetic analysis was performed to identify the sub/genotype of each sequence in the dataset (Figure S1).

### 2.2. Screening Using the Currently Approved USDA Fusion Gene rRT-PCR Assay

Fifty APMV-1 isolates from SEPRL were selected as a representative set of all 21 APMV-1 class II genotypes (as class I has only one example of a virulent virus), excluding genotypes VIII (extinct) and XI (only detected on Madagascar and not identified during the last decade) (Table 1). RNA was isolated by using the MagMAXTM 96 AI/ND viral RNA isolation kit (Ambion, Austin, TX, USA) with a KingFisher (ThermoFisher Scientific, Waltham, MA, USA) magnetic particle processor. These viruses were grown in specific-pathogen-free embryonating chicken eggs [32] and were expected to produce low cycle threshold (Ct) values. To establish that the viruses were of a high titer, they were first tested with the sensitive USDA-approved screening assay, which targets a conservative region of the matrix gene of APMV-1 [11]. Using the AgPath-IDTM One-Step RT-PCR reagents (Applied Biosystems ^TM^, Foster Ciry, CA, USA) with a 25 µL reaction volume, samples were run on a 7500 Fast Real-time PCR system (Applied Biosystems^TM^, Foster Ciry, CA, USA) with an initial reverse transcription step at 45 °C for 10 min, an initial denaturation step at 95 °C for 10 min, and 40 cycles of 94 °C for 10 s, 56 °C for 30 s, and 72 °C for 10 s.

Additional screening was performed with the current F-gene assay to establish the baseline detection sensitivity against the representative sample set. Samples were run on the 7500 Fast Real-time PCR system (Applied Biosystems^TM^, USA) with an initial reverse transcription step at 45 °C for 10 min, then an initial denaturation step at 95 °C for 10 min, and 40 cycles at 95 °C for 10 s, 58 °C for 30 s, and 72 °C for 10 s using AgPath-IDTM One-Step RT-PCR reagents (Applied Biosystems, USA).

### 2.3. Primer Design and In Silico Analyses

Single nucleotide polymorphism (SNP) analysis—to identify the proportions of polymorphisms found at every nucleotide position—was utilized for the class II virulent APMV-1 sequence database. The SNP analysis was performed utilizing the paramyxovirus section of the Virus Pathogen Resource (ViPR) [33]. This approach scored each nucleotide position for SNP conservation (0 = 100% conserved and 200 = equal percentage of each nucleotide) and then, using rolling boxcar average for 20 nucleotides sections, identified the most conserved areas of fusion gene to select regions suitable for primer and probe design. The alignment was also manually inspected for optimal selection of primers and probe to avoid regions with runs of nucleotides, and to target regions with balanced AT/GC ratio and as conserved as possible 3′ nucleotides [12]. The areas of lower SNP scores were considered optimal targets for primer selection. Primers were designed with Primer3 [34] implemented in Geneious Prime 2022.2.2 for the main virulent APMV-1 sub-genotypes that were determined to have lower detection sensitivity by the currently approved virulent F-gene APMV-1 assay [11,23]. These sub/genotypes were defined as those with ≥3-log difference between the M- and F-gene rRT-PCR assays. The newly designed oligonucleotides were quality control assessed for primer dimers formation, hairpin formation, and melting temperatures using Geneious Prime.

### 2.4. Real-Time Reverse Transcription Polymerase Chain Reaction (rRT-PCR)

Following screening with the current F-gene assay, the set of samples was used to optimize the newly designed assay for improved detection. All samples were run using the 7500 Fast Real-time PCR system (Applied Biosystems^TM^, USA) with an initial reverse transcription step at 45 °C for 10 min, then an initial denaturation step at 95 °C for 10 min, and 40 cycles at 95 °C for 10 s, 58 °C for 30 s, and 72 °C for 10 s using Path-IDTM Multiplex One-Step RT-PCR (Applied Biosystems^TM^, USA) reagents. An exogenous internal positive control (XIPC) [29] was added to the new assay (multiplex APMV-1 F-gene assay used hereafter). The multiplex F-gene assay included a 25× primers–probes mix (25× PPM) of the original forward primers and probe, along with a new forward primer (F+4835_48F) and two additional probes (F+4870_XIV_FAM and F-4894_VII_FAM) using the final concentration listed in Table 2. The following amounts per reaction were used: 12.5 μL of multiplex RT-PCR buffer, 2.5 μL of multiplex enzyme mix, 1 μL of 25× PPM (multiplex), 1 μL of the 25× XIPC PPM, and 8 μL of purified RNA for a total reaction volume of 25 μL.

### 2.5. Assay Verification

For analytical sensitivity, limit of detection (LOD) was performed with ten-fold serial dilutions for both the current and multiplex F-gene assays. For analytical specificity, near-neighbor testing was performed with strains of APMV serotypes 2–10 and 13. Infectious bronchitis virus (IBV) and influenza A virus (IAV) were tested as representative of other chicken respiratory pathogens. Clinical field samples were tested using the new F-gene assay across 5 different locations, including the Texas A&M Veterinary Medical Diagnostic Laboratory (TVMDL) locations (College Station, Canyon, Center, and Gonzales) and SEPRL. At all 5 locations, clinical field samples from various sources, such as swabs, multiple types of tissues (trachea, feces, brain, kidney, intestines, and choana), and the environment were evaluated with the new assay. APMV-1 panel samples were examined with the new multiplex assay at three of the TVMDL locations. Additional samples available at SEPRL, including known positive swabs collected from experimentally infected birds, and FTA cards from Guatemala and Mexico, were also used to verify the new multiplex assay.

## 3. Results

### 3.1. Database Curation

A total of 3164 complete F-gene sequences of class II were utilized in the final curation of the database (Table S1). A total of 1008 F-gene sequences were removed from the database based on the following criteria: duplicate sequences (n = 454), recombinant forms (n = 80), vaccine (n = 82), vaccine escape sequences (n = 324), partial fusion sequence (n = 11), or other incomplete sequences (n = 57). This resulted in a final representative curated dataset of 2206 class II F-gene sequences, 2016 of which met the virulence criterion of the World Organisation for Animal Health.

### 3.2. rRT-PCR Assay Development and In Silico Analysis

To establish the sensitivity of the current fusion gene assay, RNA from representative APMV-1 isolates were assessed using the current APMV-1 M- and F-gene assays (Table 3). The matrix gene assay demonstrated high viral load in all genotypes harboring viruses virulent in poultry (Ct between 9.8 and 17.4, with the exception of one isolate of sub-genotype XIII.2.1 for which the matrix gene assay is known to have low sensitivity [35]). The current fusion gene assay results demonstrated comparable sensitivity for most genotypes. Expectedly, the fusion gene assay did not detect viruses of low virulence. However, it was determined that the fusion gene assay did not detect or detected with lower sensitivity three sub/genotypes (Table 3). Sub-genotypes VII.1.1 and VII.2 fell into this category with ≥3-log difference between matrix and current fusion gene rRT-PCR results. Additionally, the tested genotype XIV isolates were not detected by the current assay, even though they had an average Ct value of 16.25 using the matrix gene assay. For most of all other virulent viruses, the current F-gene assay produced Ct values comparable to those of the matrix gene (or approximately within a log).

Output from the SNP analysis aided in designing primer and probes to address the issues of the sub-genotypes with lower detection sensitivity for the current assay. The analyses demonstrated that the genetic diversity within class II virulent APMV-1 isolates is high, which is likely the reason for the current USDA-approved virulent assay to miss or have decreased sensitivity with certain sub/genotypes. SNP in silico showed similar results to the primer analyses and suggested that the main mismatches between the current assay and the genotypes for which detection is lacking or is sub-optimal are in the probe (SNP index average of 26.6) and forward primer (SNP index average of 23.9) (Table S2).

New primers and probes were designed in silico and added to the current USDA-approved fusion gene assay. Ultimately, the new primers and probes provided efficient detection of sub-genotypes VII.1.1, VII.2, and XIV (Table 3 and Table S3). All newly designed primers and probes were verified individually prior to being combined in the newly designed fusion gene multiplex assay. Sensitive detection of the tested genotype XIV isolates was achieved by using the newly designed probe, F+4870_XIV_FAM (Table S4). The sensitivity of detection for genotype XIV was evaluated as excellent with Ct values ranging from 16 to 19 (5 replicates), compared to no detection (Ct = 40) with the original assay (Table S4). The sensitivity of detection for the viruses of sub-genotypes VII.1.1 was improved with Ct values ranging from 13 to 17 for different isolates (3 replicates) compared to Ct values of 29 to 35 when the original assay was utilized. Sensitive detection of sub-genotype VII.1.1 isolates was achieved by using the newly designed probe, F-4894_VII_FAM (Table S4). The sensitivity of detection for the viruses of sub-genotypes VII.2 was improved with Ct values ranging from 18 to 19 for different isolates (2 replicates) compared to Ct values of 24 to 31 when the current assay was utilized. This sensitive detection of genotype VII.2 isolates was achieved by using the newly designed primer, F+4835_48F (Table S4).

Following the individual validation of the new primer/probes mentioned above, they were tested in combination to verify the efficiency of the assay as a multiplex using the Path-ID™ Multiplex One-Step RT-PCR kit. Under these conditions, the Ct values of the sub-genotypes VII.1.1, VII.2, and XIV performed as well as the newly designed primers and probes on an individual basis and showed much improved detection compared to the current USDA-approved assay (Table 3). The sensitivity for the other sub/genotypes was maintained with the new multiplex F-gene assay compared to the current F-gene assay (Table 3 and Table S5).

### 3.3. rRT-PCR Assay Verification

To establish a limit of detection (LOD) for the new multiplex assay, ten-fold serial dilutions were performed using APMV-1 of three different genotypes (Table S6). The LOD was determined to be 10^2.4^ EID_50_, 10^2.1^ EID_50_, and 10^2.5^ EID_50_ per mL for virus genotypes V.2, VII.1.1, and XII, respectively (Table S6). A decrease in the LOD compared to the original assay was observed in genotypes VII.1.1 (10^5.1^ EID_50_) and XII (10^3.5^ EID_50_) and the LOD was maintained for genotype V.2 (Table S6). No near-neighbor detection was observed for the new multiplex assay for all tested strains of APMV (serotypes 2–10, 13), as well as for IBV and AIV.

The multiplex assay was validated with 406 clinical samples at five different laboratories using different sample sources (Table S7). The new assay did not produce any non-specific amplification or detection in nine out of ten runs. In one run, two swab samples with unknown APMV-1 status tested at SEPRL showed positive results. The tentatively positive samples were retested with the original fusion gene assay. While one sample tested negative, the other one showed a positive result when tested in duplicate (Table S7). The use of diverse sets of samples did not affect the assay and demonstrated it is fit-for-purpose. Among the clinical samples tested, 17 of these samples were also found to have infectious bronchitis virus RNA (Table S7), which is further demonstration that the test does not detect other respiratory viruses that may be present in a sample. None of the clinical samples had IAV.

Further validation was performed on retained experimental positive samples collected from experimentally infected birds, FTA cards, and other known positive samples (n = 34, n = 29, and n = 75, respectively) (Tables S8–S10). Of the retained experimental positive samples (n = 30), all but one were detected in both replicates, and the remaining sample was detected in only one of the replicates (Table S8). All the positive experimental samples were also tested using the screening L-gene SEPRL assay [12]. This assay has an LOD that is very close to the LOD of the new multiplex assay used here and provides good means for comparison on assay efficiency. Among the 30 FTA cards test, two FTA cards with detection using the new assay were confirmed positive using the original assay (Table S9). These two samples were also analyzed using next-generation sequencing and had APMV-1 reads and were confirmed as virulent APMV-1. Finally, the known positive samples with expected Ct values established with the current assay were tested with the new multiplex fusion gene assay at three different locations (Table S10). All the results fell within the expected range and among these, the majority the Ct values were at the lower end of the expected range and some Ct values were below the expected lower end (Table S10). Importantly, the results demonstrated the consistency of the new multiplex assay in a range of testing locations (Table S10).

## 4. Discussion

Virulent APMV-1 strains are circulating worldwide and continue to be a threat to the poultry industry. Continuous monitoring and surveillance of suspect cases using sensitive and specific assays is critical. However, the current and most widely used F-gene assay has been shown to have decreased sensitivity or false negative results for several currently circulating genotypes. For these reasons, the verification of an improved assay is of the utmost importance. Through the globally increasing sequencing efforts and the use of next-generation sequencing, there are considerably more sequence data available, which allows comprehensive sequence analyses of APMV-1 genetic diversity to improve the detection efficiency of molecular assays. Here, we describe an updated and validated rRT-PCR assay that detects all known and circulating APMV-1 chicken velogenic genotypes. In addition to improving upon the current assay, an exogenous internal positive control was incorporated into the new multiplex rRT-PCR assay. Validation was performed at five different laboratories using 544 samples including swabs and tissues collected from clinical samples and samples with a known status (Tables S6–S8 and S10), thereby demonstrating the reproducibility of the new multiplex assay.

The currently used F-gene assay does not detect all APMV-1 sub-genotypes, demonstrated by the lower detection sensitivity or lack of detection for sub/genotypes V.1, VII.1.1, VII.2, and XIV from cultured high titer viruses (Table 2). This phenomenon of decreased assay sensitivity is unsurprising due to how rapid viral evolution occurs, especially with RNA viruses and APMV-1 in particular [1,2,36]. Furthermore, the widespread use of only partially matched live vaccine strains circulating with other genotypes may increase the diversification of APMV-1 through positive selection and this may contribute to the decreased accuracy of the current F-gene assay [37]. The currently used fusion gene assay was designed in response to the APMV-1 outbreak in California during 2002–2003 and was validated predominantly using strains belonging to the “early” genotypes II–VII [10,11]. Bioinformatics analysis was also constrained by what sequences were available at the time, and since 2003, multiple novel genotypes have been described. Some APMV-1 strains are only circulating on certain continents and geographical locations; however, due to increased globalization and trade, these pose a threat to the poultry industries worldwide. It is therefore important for laboratories to maintain preparedness with reliable assays that can detect all circulating strains.

The newly developed multiplex F-gene rRT-PCR assay provides sensitive and specific identification of the currently known and circulating virulent APMV-1. Development of an assay that targets all sub/genotypes is challenging due to the high variability region of the fusion gene where the cleavage site resides as observed through our SNP analyses (Table S1). The development of an assay targeting more conserved regions on the fusion genes was deemed not feasible due to the requirement of detection of virulent APMV-1 strains via the cleavage site as the primary virulence determinant. Thus, a multiplex assay was developed to achieve detection of all currently circulating chicken velogenic genotypes, and specifically to improve detection of sub/genotypes VII.1.1, VII.2, and XIV. Using a comprehensive F-gene sequence dataset, SNP and in silico analyses resulted in the addition of a primer and two probes to be used concurrently with the existing assay. With the variability surrounding the F-gene cleavage site in virulent APMV-1, it can be challenging to combine multiple primers and probes into one assay. To help mitigate this, we describe the use of a 25× PPM for the master mix. This allows more accurate handling, minimizes errors for multiplex assays with multiple primers and probes, and ensures increased consistency between assays. Fortin and co-authors developed a new array of three separate rRT-PCR assays that enable the identification of virulent and avirulent APMV-1 (including APMV-1 of class I) in a dual mode [38]. Other assays have tried to address the issue of variability by adding numerous indeterminate bases to the primers and probes from the original assay to maintain a singleplex assay [11,16]. Although our multiplex assay has several primers and probes, assay sensitivity was improved by utilizing a more advanced one-step RT-PCR kit designed for such needs.

To monitor for any potential issues with PCR inhibitors and amplification, an exogenous internal positive control (XIPC) was implemented into the new F-gene multiplex assay during the validation process [23]. This internal positive control aids in the detection of false negatives, specifically when added at the nucleic acid extraction step [29]. Within each sample, the expectation is for the XIPC to amplify, thus confirming that there was effective nucleic acid extraction and the PCR reagents functioned as expected. The XIPC was also added to the individual assays for genotype VII and genotype XIV developed during the design of the multiplex assay, in case these need to be used independently (Table S2). The addition of an XIPC is an expectation of new molecular tests adopted for use by the NAHLN. Although the typical testing workflow is to first screen using the matrix-gene assay to identify any class II AMPV-1, which should ideally have an XIPC, in some circumstances where an outbreak in an area has been confirmed as virulent NDV and the fusion test is working as expected, primary screening may be performed with F-test to increase the speed of the final diagnosis. In this scenario, having an XIPC is strongly desired to reduce false negatives from inhibitors or test failure.

The use of multiple probes in a single rRT-PCR assay can be advantageous but can also have some limitations due to non-specific or background amplification. These were observed for a few samples using the new multiplex F-gene assay when multiple probes were combined into a single reaction. It is believed this can be a result of probe interactions or non-specific binding (Figure S2). To avoid the identification of false positives, samples with higher Ct values or abnormal ΔRn curves should have their ΔRn and component plots examined. Background amplification in a multiplex assay was attributed to the non-specific binding of the probe and was reduced by increasing the annealing/extension temperature and adjusting the final probe concentration [39]. Another group suggested that the non-specific amplification at higher Ct values could be due to PCR inhibitors or target degradation, and suggested the importance of examining the PCR reagents, nucleic acid quality, and the selection of fluorescence reporter dyes [40]. Our addition of the XIPC in the multiplex can aid in the detection of PCR inhibitors and nucleic acid extraction quality. Nevertheless, the importance of screening the ΔRn and component plots for suspect samples is highlighted.

In summary, we describe an improved multiplex F-gene rRT-PCR assay for the detection of virulent APMV-1. Our findings demonstrate the importance of routine evaluation of rRT-PCR assays to account for possible diversification of the APMV-1 fusion gene which may result in lower sensitivity and specificity over time. The increased availability of F-gene sequences from around the world and improved PCR chemistry kits facilitated the design of a new forward primer and two probes which were added to the current F-gene assay resulting in an optimized multiplex tool detecting currently circulating virulent APMV-1. Using several laboratories for assay validation and a large array of sample types, we demonstrated the improved multiplex assay to be robust and accurate in the detection of APMV-1 with the added benefit of an internal control to screen for PCR inhibitors and extraction efficiency.

## Figures and Tables

**Table 1:** AMPV-1 isolates (n = 50) selected as a representative sample set of the 21 current circulating genotypes used in the development of the fusion gene multiplex rRT-PCR assay. Isolates labeled as being of “variant” virulence were pigeon and cormorant antigenic and genetic variants which have variable, predominantly mild virulence in chickens.

Study ID	Isolate ID	Genotype	Old Genotype	Species	Country	Year	Cleavage Site Motif	Virulence
1	BS/350 (N35)	I.1.1	I a	Chicken	Bassa, Nigeria	2009	RKQGRL	Low
2	APMV-t-AN-3201110 (K4)	I.2	I b	Ruddy Shelduck	Askania-Nova, Ukraine	2010	GKQGRL	Low
3	Askania-Nova/72-28-03	I.2	I b	White-Fronted Goose	Askania-Nova Reserve, Ukraine	2013	GKQGRL	Low
4	P/AV/FL/475985/07	I.2	I b	Avian	Florida, USA	2007	GKQGRF	Low
5	ZOOMAT-10-08	I.1.2.1	I c	Plain Chachalaca	Mexico	2009	GKQGRL	Low
6	TK 80136	II	II	Chicken	Georgia, USA	2009	GRQGRL	Low
7	LaSota	II	II	Chicken	USA	1946	GRQGRL	Low
8	NDV/pigeon/NovoSelo/1995	III	III	Pigeon	Novo Selo, Bulgaria	1995	RRQRRF	Virulent
9	SPVC/Karachi/NDV/1	III	III	MukteswarVvaccine (SPVC)	Karachi, Pakistan	1974	RRQRRF	Virulent
10	NDV/chicken/Haskovo/1968	IV	IV	Chicken	Haskovo, Bulgaria	1968	RRQRRF	Virulent
11	Kano/1973 (N52)	IV	IV	Chicken	Nigeria	1973	RRQRRF	Virulent
12	A00520441/442 (MN1)	XIX	V a	Double-Crested Cormorant	Minnesota Lake, MN, USA	2008	KRQKRF	Variant
13	A00841381	XIX	V a	Double-Crested Cormorant	Portland, ME, USA	2010	KRQKRF	Variant
14	A00874288	XIX	V a	Double-Crested Cormorant	Barnstable County, MA, USA	2010	KRQKRF	Variant
15	NDV/chicken/Furen/1988	V.1	V b	Chicken	Furen, Bulgaria	1988	RRQKRF	Virulent
16	P/CK/Belize/4224-3/08	V.1	V b	Chicken	Spanish Lookout, Cayo, Belize	2008	RRQKRF	Virulent
17	00-448-13	V.1	V b	Chicken	Honduras	2000	RRQKRF	Virulent
18	NC/23/11	V.2	V c	Chicken	Aguascalientes, Mexico	2011	RRQKRF	Virulent
19	Mbeya/MT15	V.3	V d	Chicken	Tanzania	2012	RRQKRF	Virulent
20	TX 3988 (D1548)	VI.2.1.1.1	VI a	Eurasion Collared Dove	Houston, TX, USA	2004	RRKKRF	Variant
21	ND0007187	VI.2.1.1.1	VI a	Rock Pigeon	Allegheny, PA, USA	2013	RRKKRF	Variant
22	TX 6295 (D0729)	VI.2.1.1.1	VI a	Eurasion Collared Dove	Houston, TX, USA	2006	RRKKRF	Variant
23	NDV/fowl/DolnoLinevo/1992	XX	VI c	Chicken	Dolno Linevo, Bulgaria	1992	RRQKRF	Virulent
24	APMV-p-Kh-230113 (K13)	XXI.1.1	VI g	Pigeon	Kharkiv, Ukraine	2013	KRQKRF	Variant
25	VRD08/385 (N23)	XXI.2.1.2	VI h	Quail	Nigeria	2008	RRRKRF	Variant
26	Pk25	XXI.1.2	VI m	Pigeon	Lahore, Punjab, Pakistan	2015	RRQKRF	Variant
27	PKR/CK/15	VII.2	VII i	Chicken	Sheikhupura, Punjab, Pakistan	2015	RRQKRF	Virulent
28	sample 120	VII.2	VII i	Chicken (Broiler Breeders)	Beer-Tuvia, Israel	2013	RRQKRF	Virulent
29	NDV/EG/CK/104/12	VII.1.1	VII j	Chicken (Broiler)	Qena, Egypt	2012	RRQKRF	Virulent
30	NDV/EG/CK/136/12	VII.1.1	VII b	Chicken (Broiler)	Qena, Egypt	2012	RRQKRF	Virulent
31	ZJ1	VII.1.1	VII d	Goose	China	2000	RRQKRF	Virulent
32	Kardam	VII.1.1	VII d	Chicken	Bulgaria	2008	RRQKRF	Virulent
33	78	VII.1.1	VII e	Duck	Long Bien, Vietnam	2002	RRQKRF	Virulent
34	NDV04-23 (C12)	IX	IX	Chicken	China	2004	RRQRRF	Virulent
35	P/TY/MN/77/08	X.1	X a	Turkey	Minnesota, USA	2008	RKQGRF	Low
36	MN00-39	X.2	X b	Juvenile Mallard	Minnesota, USA	2000	EKQGRL	Low
37	P/TY/MN/4661/09	X.1	X a	Turkey	Minnesota, USA	2009	RKQGRF	Low
38	TX01-130	X.1	X a	Mottled Duck	Brazoria County, TX, USA	2001	GKQGRL	Low
39	P\avian\peru\1918-03\08	XII.1	XII a	Chicken	Peru	2008	RRQKRF	Virulent
40	Tanga/N38	XIII.1.1	XIII a	Chicken	Tanzania	2012	RRQKRF	Virulent
41	Tanga/N1	XIII.1.1	XIII a	Chicken	Tanzania	2012	RRQKRF	Virulent
42	SPVC/Karachi/NDV/27	XIII.2.1	XIII b	Chicken	Karachi, Pakistan	2006	RRQKRF	Virulent
43	KT/MSH/15C (N2)	XIV	XIV b	Pigeon	Katsina, Nigeria	2009	RRRKRF	Virulent
44	VRD09/546 (N4)	XIV	XIV b	Golden Eagle	Taraba, Nigeria	2009	RRRKRF	Virulent
45	P/Chicken/FO-DR/499-31/08	XVI	XVI	Chicken	Dominican Republic	2008	RRQKRF	Virulent
46	Kudu-113/1992 (N56)	XVII	XVII anc	Duck	Nigeria	1992	RRQKRF	Virulent
47	ZM/KN/GF01bC (N6)	XVII.1	XVII a	Guinea Fowl	Zamfara, Nigeria	2009	RRQKRF	Virulent
48	VRD17/04 (N2)	XVII.1	XVII a	Quail	Nigeria	2004	RRQKRF	Virulent
49	228-7	XVII.2	XVII b	Chicken	Nigeria	2006	RRQRRF	Virulent
50	OOT/4/1 (N69)	XVIII.2	XVIII b	Chicken	Ota, Nigeria	2009	RRQKRF	Virulent

**Table 2:** Primers and probes designed for the fusion gene multiplex rRT-PCR assay, including those part of the original fusion gene assay.

Oligos	Type	5′-3′ Sequence	bp	Concentration (μM)	Source
F+4829	Fwd Primer	GGTGAGTCTATCCGGARGATACAAG	25	0.384	Creelan et al., 2002 [23]
F-4939	Rev Primer	AGCTGTTGCAACCCCAAG	18	0.192	Wise et al., 2004 [11]
F (VFP-1)-4894_FAM	Probe	AAGCGTTTCTGTCTCCTTCCTCCA	24	0.0624	
F+4870_XIV_FAM	Probe	TGGAGGAAGACGACGGAAACGTTT	24	0.0624	THIS STUDY -MULTIPLEX ASSAY
F+4835_48F	Fwd Primer	TCCATCCGCAAGATCCAAGG	20	0.192	
F-4894_VII_FAM	Probe	AARCGTTTTTGTCTCCTTCCTCCG	24	0.0672	

**Table 3:** Results from the original matrix and fusion gene rRT-PCR assays for the 50 APMV-1 samples.

Study ID	Genotype (Old Genotype)	M-Gene Assay Ct Value	Original F-Gene Assay Ct Value	Multiplex F-Gene Assay Ct Value (Average)	Multiplex Assay F-Gene Ct Value (Median)	XIPC Ct Value (Average)	XIPC Ct Value (Median)
1	I.1.1 (Ia)	13.0	40.0	37.7	40.0	31.4	31.0
2	I.2 (Ib)	11.9	40.0	34.7	40.0	34.1	31.2
3	I.2 (Ib)	14.8	40.0	40.0	40.0	31.1	31.2
4	I.2 (Ib)	17.9	40.0	40.0	40.0	34.3	33.9
5	I.1.2.1 (Ic)	15.7	40.0	40.0	40.0	30.8	30.9
6	II (II)	10.3	40.0	40.0	40.0	31.9	32.0
7	II (II)	9.9	40.0	40.0	40.0	31.6	31.3
8	III (III)	18.3	20.9	21.2	21.3	31.8	31.0
9	III (III)	19.2	21.9	24.0	24.5	30.9	31.0
10	IV (IV)	16.5	21.6	24.3	24.1	31.2	31.2
11	IV (IV)	10.3	15.1	19.5	17.5	35.3	35.2
12	XIX (Va)	20.0	40.0	40.0	40.0	31.2	31.2
13	XIX (Va)	21.2	40.0	38.9	40.0	30.9	30.9
14	XIX (Va)	19.8	40.0	38.8	40.0	30.8	30.8
15	V.1 (Vb)	14.6	17.6	21.8	23.0	30.7	30.9
16	V.1 (Vb)	12.8	12.8	14.7	14.8	31.4	31.1
17	V.1 (Vb)	12.6	27.1	28.1	29.6	30.8	30.9
18	XX (Vc)	11.8	15.3	17.0	16.8	30.5	30.4
19	V.3 (Vd)	12.2	20.4	22.9	23.1	30.3	30.4
20	VI.2.1.1.1 (VIa)	20.3	40.0	40.0	40.0	31.9	32.2
21	VI.2.1.1.1 (VIa)	25.3	40.0	40.0	40.0	32.7	31.1
22	VI.2.1.1.1 (VIa)	17.6	40.0	39.6	40.0	31.3	31.2
23	XX (VIc)	12.5	14.9	18.2	17.2	31.5	30.9
24	XXI.1.1 (VIg)	18.5	40.0	40.0	40.0	31.4	31.4
25	XXI.2.1.2 (VIh)	16.8	40.0	40.0	40.0	31.8	32.1
26	XXI.1.2 (VIm)	17.4	23.2	30.8	26.7	33.6	30.5
27	VII.2 (VIIi)	11.4	22.3	22.1	22.4	30.7	30.7
28	VII.2 (VIIi)	12.1	25.2	22.7	22.4	30.8	31.1
29	VII.1.1 (VIIj)	9.8	25.9	22.0	19.6	30.9	31.1
30	VII.1.1 (VIIb)	14.1	30.5	25.2	22.4	31.3	31.4
31	VII.1.1 (VIId)	9.8	19.0	16.9	16.0	31.7	32.1
32	VII.1.1 (VIId)	12.6	18.9	17.6	17.3	30.4	30.7
33	VII.1.1 (VIIe)	10.0	17.2	15.5	14.0	30.7	30.7
34	IX (IX)	13.8	18.6	17.5	17.4	31.4	32.1
35	X.1 (Xa)	14.9	40.0	40.0	40.0	33.9	33.2
36	X.2 (Xb)	13.9	40.0	40.0	40.0	38.1	38.2
37	X.1 (Xa)	14.4	40.0	37.7	40.0	33.1	33.2
38	X.1 (Xa)	12.2	40.0	40.0	40.0	35.7	34.0
39	XII.1 (XIIa)	11.3	17.0	18.1	18.6	30.8	31.0
40	XIII.1.1 (XIIIa)	17.0	15.8	20.5	22.0	31.5	31.6
41	XIII.1.1 (XIIIa)	16.4	15.2	18.0	18.6	30.6	30.7
42	XIII.2.1 (XIIIb)	26.1	19.8	22.5	22.7	29.9	30.1
43	XIV (XIVb)	15.1	40.0	24.2	17.1	30.5	30.3
44	XIV (XIVb)	17.4	40.0	24.4	17.8	31.2	30.9
45	XVI (XVI)	11.6	15.0	18.2	17.5	31.1	31.1
46	XVII (XVIIanc)	13.3	17.2	20.5	19.5	31.2	30.4
47	XVII.1 (XVIIa)	13.5	14.8	17.9	17.3	30.9	30.6
48	XVII.1 (XVIIa)	12.3	15.1	18.4	17.6	30.8	30.6
49	XVII.2 (XVIIb)	15.7	17.6	20.8	20.0	30.8	30.8
50	XVIII.2 (XVIIIb)	15.2	22.6	26.6	27.3	30.7	31.0
NTC		40.0	40.0	40.0	40.0	NA	NA
PAC (no XIPC added)		23.5	24.2	25.7	25.7	NA	NA

NTC = no template control, PAC = positive amplification control, NA = not applicable.

## Data Availability

All GenBank accessions for the complete F-gene sequences from the 50 APMV-1 strains are available in Table S4.

## Supplementary Materials

The following supporting information can be downloaded at: https://www.mdpi.com/article/10.3390/v17050670/s1, Figure S1: Phylogenetic tree of APMV-1 fusion gene sequences in curated dataset used in this study; Figure S2: Plots of background signal observed with the multiplex assay; Table S1. Complete curated APMV-1 class II fusion gene database; Table S2. ViPR SNP analysis output to assess the binding regions of the newly designed primer and probes; Table S3. Newly designed primer and probes concentration for AgPath-ID One-Step RT-PCR reagents and the thermal profile conditions for the genotype VII probe, XIV, probe, VII forward primer, and combination of XIV+VII probes; Table S4. Individual assessment of newly designed primer and probes compared to the original F-gene rRT-PCR assay; Table S5. Comparison of the new F-gene multiplex rRT-PCR assay with the original F-gene assay; Table S6. Limit of detection for the new F-gene multiplex assay compared to the original F-gene assay, performed on three AMPV-1 genotypes; Table S7. Clinical samples tested with new F-gene multiplex rRT-PCR assay at 5 different laboratories with inclusion of the XIPC; Table S8. Testing of the new F-gene multiplex on known positive samples from experimental challenge; Table S9. Testing the new F-gene multiplex on FTA cards obtained from Mexico at SEPRL; Table S10. Evaluation of the F-gene multiplex rRT-PCR assay with known positive and known negative samples at four different laboratories.

## Abbreviations

The following abbreviations are used in this manuscript:

APMV-1	Avian paramyxovirus 1
ND	Newcastle disease
rRT-PCR	Real-time reverse transcriptase PCR
LD	Linear dichroism
NP	Nucleoprotein
M	Matrix
F	Fusion
L	Large RNA polymerase
NAHLN	National Animal Health Laboratory Network
XIPC	Exogenous internal positive control
MAFFT	Multiple Alignment with Fast Fourier Transformation
SEPRL	Southeast Poultry Research Laboratory
SNP	Single nucleotide polymorphism
ViPR	Virus Pathogen Resource
25X PPM	25X primers–probes mix
LOD	Limit of detection
IBV	Infectious bronchitis virus
IAV	Influenza A virus
TVMDL	Texas A&M Veterinary Medical Diagnostic Laboratory
NTC	No template control
PAC	Positive amplification control
