# Supplementary figures and images for: Optimization and Validation of Universal Real-Time RT-PCR Assay to Detect Virulent Newcastle Disease Viruses
Source: Viruses. 2025 May 3;17(5):670. doi: 10.3390/v17050670 (PMC12115569; doi:10.3390/v17050670)

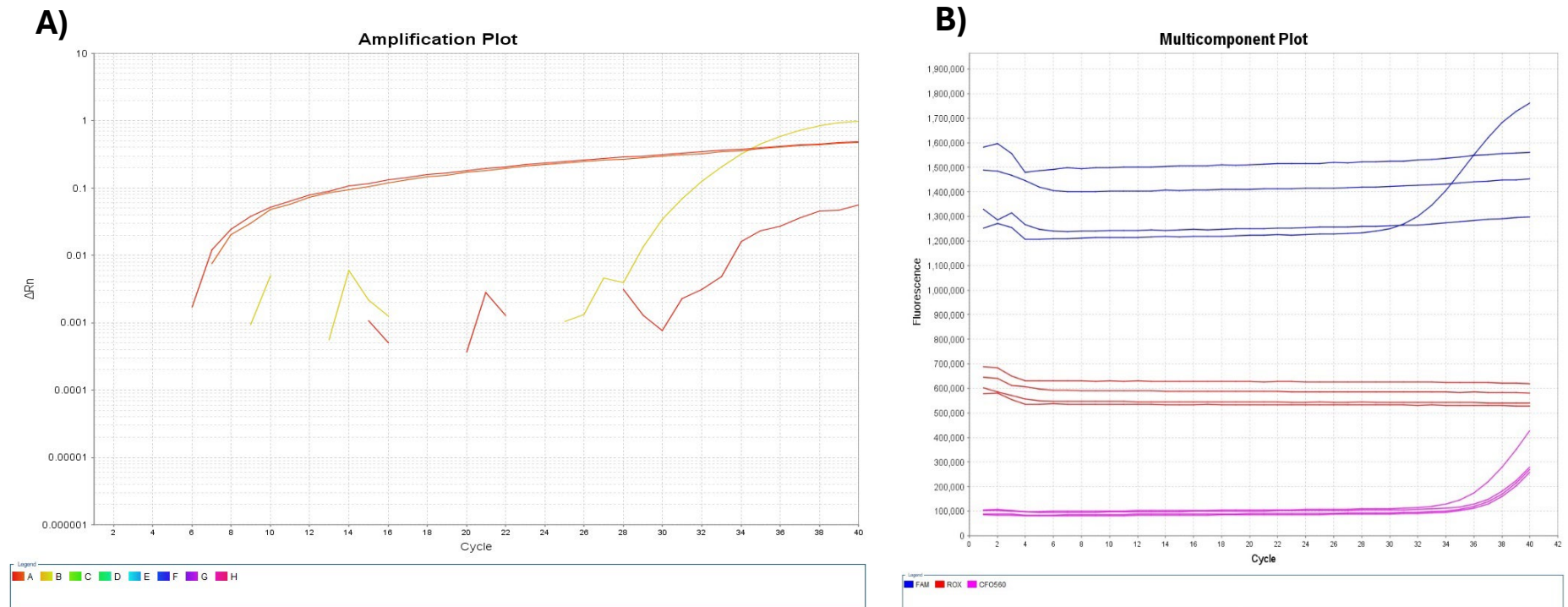

Supplement: Supplementary file 1 [file viruses-17-00670-s001.zip › Supplemental_Material/Fig_S2.pdf]
