# Supplementary material for: Optimization and Validation of Universal Real-Time RT-PCR Assay to Detect Virulent Newcastle Disease Viruses
Source: Viruses. 2025 May 3;17(5):670. doi: 10.3390/v17050670 (PMC12115569; doi:10.3390/v17050670)
Supplement: Supplementary file 1 [file Fig_S1.pdf › Supplemental_Material/Fig_S1.pdf]

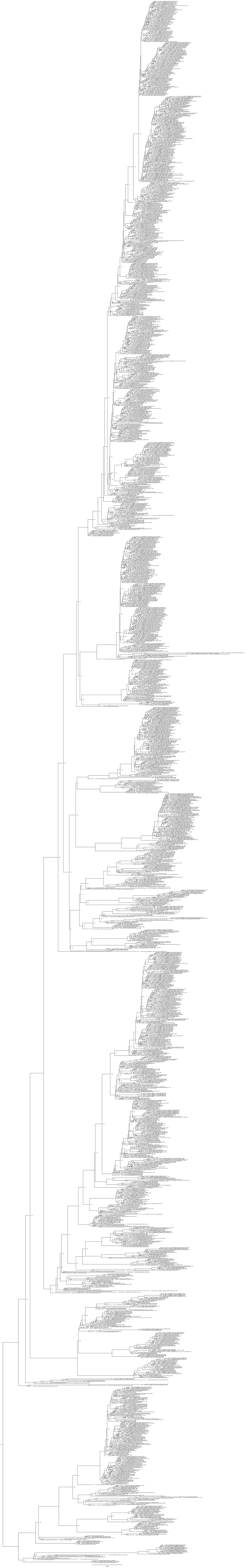
0.03
